# Erector Spinae Muscle Activation During Forward Movement in Individuals With or Without Chronic Lower Back Pain: A Systematic Review and Meta-analysis

**DOI:** 10.1016/j.arrct.2023.100280

**Published:** 2023-07-14

**Authors:** Euan W. Taylor, U. Chris Ugbolue, Yang Gao, Yaodong Gu, Julien S. Baker, Frédéric Dutheil

**Affiliations:** a Faculty of Sports Science, Ningbo University, Ningbo, Zhejiang Province, China; b School of Health and Life Sciences, Institute for Clinical Exercise & Health Science, University of the West of Scotland, South Lanarkshire, United Kingdom; c Centre for Health and Exercise Science Research, Department of Sport, Physical Education and Health, Hong Kong Baptist University, Kowloon Tong, Hong Kong; d CNRS, LaPSCo, Physiological and Psychosocial Stress, University Hospital of Clermont-Ferrand, CHU Clermont-Ferrand, Preventive and Occupational Medicine, WittyFit, Université Clermont Auvergne, Clermont-Ferrand, France

**Keywords:** Back muscle, Back pain, Electromyography, Rehabilitation

## Abstract

**Objective:**

To investigate the differences between erector spinae muscle activation in healthy individuals and patients with Chronic Lower Back Pain (CLBP) by conducting (a) systematic review and (b) meta-analysis.

**Data Sources:**

PubMed, ScienceDirect, SPORTDiscus, and Google Scholar were used to conduct the searches, which included studies up to the 31st of March 2023 with no start date specified.

**Study Selection:**

Any study otherwise meeting eligibility criteria was included if it reported either (1) a standard mean difference effect size; or (2) the means, SDs, and sample sizes for both the patient group and the comparator group.

**Data Extraction:**

A total of 7 case control trials were used for the systematic review and meta-analysis.

**Data Synthesis:**

The systematic review and meta-analysis revealed that total standardized mean difference in erector spinae muscle activation between healthy individuals vs patients with CLBP expressed in % maximum voluntary isometric contraction was 0.48 (95% confidence interval=0.21-0.74; *P*<.001) with the heterogeneity being I^2^=0% (*P*=.890). The electromyography (EMG) outputs showed significant differences in activation levels between the healthy and CLBP cohorts (*P*<.001).

**Conclusions:**

A small effect size was found in the meta-analysis. The muscle activation levels of the erector spinae during forward propulsion were higher in CLBP individuals compared with healthy cohorts. The findings provide more clarity about the muscles that were the focus of previous research, what procedures were used to evaluate muscular contributions and what speeds the participants were moving at during the test sessions. Given the limited methodological quality of the included studies, the findings should be interpreted with caution. Future research should evaluate the effect of other factors such as walking distance and any changes in walking surfaces and gradients (ie, non-flat surfaces).

Lower back pain is a common health problem in modern society that is likely to affect individuals at some point in their lives.^1^, ^2^, ^3^ Indeed, it is a problem that places a financial burden on the health systems of different countries^4^ and is estimated to cost the United States $100 billion a year.^5^ As a treatment, exercise has been shown to be beneficial in individuals with chronic lower back pain (CLBP).^6^^,^^7^ Specifically lumbar stabilization and walking exercises not only relieve back pain but also prevent CLBP through improving muscle endurance^8^ and normal range of motion.^9^ The erector spinae muscles stabilize and mobilize the lumbar spine^10^ while keeping the body upright during forward propulsion.^11^, ^12^, ^13^ Any sport that involves high velocities and twisting movements such as soccer and dancing can affect the role the erector spinae muscles play in stabilizing the spine. By strengthening the core muscles such as erector spinae, internal/external obliques, and the transverse abdominis, the risk of injury can be reduced considerably.

A method for assessing and measuring back muscle activity can be performed using electromyography (EMG). EMG allows muscle activation to be identified and recorded, which is an important diagnostic for detecting injuries, rehabilitation, and muscle imbalances. EMG may be non-invasive where surface EMG (sEMG) sensors are used or invasive where an intra-muscular test requiring skin penetration is performed. sEMG is popular, validated, and allows wider participation from subjects as they engage in and record muscle activities associated with both functional activities of daily living^14^ and sport related activities.^15^ In a study that investigated muscle activation of the erector spinae muscles during forward bending and squatting compared with the quadriceps, it was observed that the back muscles produced much greater muscle activation.^16^ This study was important as it was hypothesized previously that individuals with CLBP exhibit a response noted as a “Guarded movement." This can be categorized as individuals with CLBP exhibiting abnormal muscle activity during physical exercise.^17^ This is often studied in research during flexion movements. Two separate studies^18^^,^^19^ reported that subjects with CLBP showed poorer levels of muscle relaxation during flexion. It was also noted that there was abnormal muscle activity during flexion extension tasks. This was suspected to be due to the guarding mechanism in relation to the onset of pain. Studies in this area are limited and results remain inconclusive. Therefore, the need for further investigative research that provides a clearer understanding of the erector spinae musculature regarding the underlying mechanisms associated with lower back pain would be an added contribution to the current knowledge in this area.

Walking is a fundamental human movement that involves the entire body working in unison to produce locomotion. The Centre for Disease Control and Prevention states that adults should aim for 150 minutes of exercise a week and be active for 60 minutes a day.^20^ Walking is an excellent method of reaching this target and getting the physical and mental benefits it provides. The benefits of physical activity are well reported, and it has been demonstrated that physical activity can help to reduce weight, blood pressure, and prevent cardiovascular diseases.^21^^,^^22^ Multiple studies have investigated the muscle activation of the erector spinae in healthy individuals and patients with CLBP during walking.^23^^,^^24^ The research suggests that individuals with CLBP exhibit much higher levels of muscle activation.^23^^,^^24^ On the other hand, chronic back pain is often the subject of research focusing on back injury. This may be because chronic pain has a more detrimental effect on quality of life and days off in the workplace because of the severity. Despite the amount of research investigations on chronic back pain,^25^, ^26^, ^27^ there is still limited evidence that provides a clear understanding and association between erector spinae muscle activation during forward movement in individuals with CLBP. Additionally, it is still unclear which muscles were the focus of previous research, what tests were used to evaluate muscular contributions and what speed were the participants moving during testing sessions?

Therefore, the purpose of this study was to investigate the differences in erector spinae muscle activation between healthy individuals and patients with CLBP by conducting (a) systematic review and (b) meta-analysis.

## Methods

### Study design

The Preferred Reporting Items of Systematic reviews and Meta-Analyses (PRISMA) guidelines were adhered to for reporting quality. Stratified analyses were conducted to compare the differences between any differences in erector spinae muscle activation between healthy individuals and patients with CLBP.

### Eligibility criteria

The following eligibility criteria were used and only the studies that met the criteria were included (i) Full text must be available; (ii) Must be a journal article, no conference papers, abstracts and thesis; (iii) All selected studies must use a 2 group design, comparing a patient group to a healthy comparison group; (iv) Participants in the study must either be healthy adults (18-84 years old) and/or have CLBP that is not from disk herniation/disease. Specifically, their pain was nonspecific and not radicular in nature; (v) Muscle Activity must be collected in the erector spinae during forward walking/running; (vi) Must be in English; (vii) Human studies only; (viii) On flat ground and not in water; and (ix) All studies must have reported either a standard mean difference effect size or the means, standard deviations, and sample sizes for both the patient group and the comparator group. If studies have taken results at multiple speeds, the authors attempted to obtain results from the median speed or the walking speed. Case control trials were to be primarily used and were used in the systematic review and meta-analysis. The experimental design for each study is reported in the summary table.

### Information sources

PubMed, ScienceDirect, SPORTDiscus, and Google Scholar were used to conduct the searches, which included studies up to the 31st of March 2023 with no start date specified. The same key words were used for each database with parenthesis and Boolean phrases being adjusted. The following search terms were used on PubMed and SPORTDiscus: EMG OR Electromyography AND Lower Back Pain AND Erector spinae and running. Search terms on Science Direct were as follows: EMG Electromyography Lower Back Pain Erector spinae running. Google Scholar used the following search: “EMG” AND “Electromyography” AND “Lower Back Pain” AND “Erector spinae” AND “running”. Studies were also identified through additional sources such as further reading.

### Study selection

**Fig 1:**
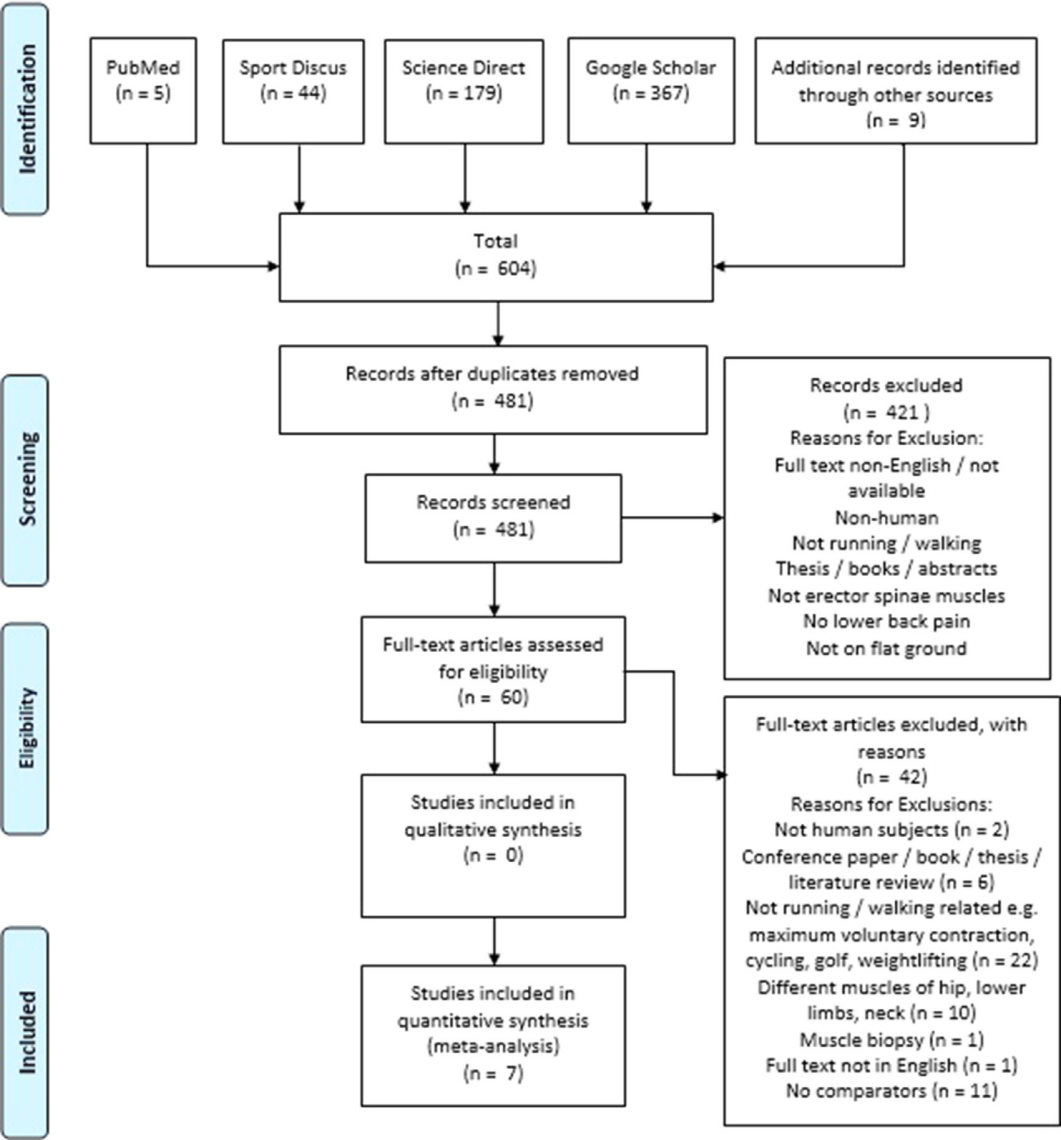
PRISMA flowchart of the systematic literature.

After collection of the results from the database searches, the author (E.W.T.) downloaded the papers to reference manager^a^ where they were organized into 1 file. Firstly, duplicates were removed from the file and then the title and abstracts were screened to remove all non-relevant studies or ones that did not meet the eligibility criteria. The authors (E.W.T., U.C.U.) then accessed the full text for each remaining study and excluded studies that were not eligible. Details of the process are outlined on the PRISMA flow diagram (fig 1). A summary of all the remaining journals used in the meta-analysis is outlined in table 1. This table identifies each study with specific characteristics such as experimental design, participants’ age and sex, participants’ height and mass, sample size, muscles used, and EMG type.

**Table 1:** Characteristics of included studies arranged in alphabetical order of author name and year of publication (Column 1)

Author (Year)	Title/Aim of the Study	Participant's Sex and Age	Participant's Height and Mass	Experimental Design	Sample Size	Walking Running Speed	EMG Type	Muscles Studied	PEDro Score
Ansari et al (2018)^28^	Lumbar muscle activation pattern during forward and backward walking in participants with and without chronic low back pain: an electromyographic study	22 men 20 women Healthy=23.52±2.44 years old CLBP=22.62±2.7 years old	Healthy=161 cm±6.5 52.72 kg±8.4 CLBP=161.1 cm±11.04 57.97 kg ±11.16	CCT	21 with CLBP 21 without CLBP	Self-selected walking speed	Surface electromyography (sEMG)	ES and M	5
Diederich (2005)^29^	Estimate of muscle contribution to spinal loads during continuous passive motion for low back pain	6 men 3 women 29-50 years old	177 cm±9.9 92.2 kg±44.2	CCT	4 with CLBP 5 without CLBP	Self-selected walking speed	sEMG	LD, M, GMA and HF	4
Farahpour et al (2018)^30^	Muscle activity and kinetics of lower limbs during walking in pronated feet individuals with and without low back pain	30 Men Healthy=26±2.9 years old CLBP=25.3±2.9 years old	Healthy=174.5cm±5.5 78.7kg±9.9 CLBP=172.8cm±4.4 79.9 kg±3	CCT	15 with CLBP and pronated feet 15 without CLBP &PF	Self-selected walking speed	sEMG	ES,TA, GA, BF, VL, GM, RA, EO, and IO	5
Hanada et al (2011)^31^	A comparison of trunk muscle activation amplitudes during gait in older adults with and without chronic low back pain	8 men 10 women Healthy=64.9±8.8 years old CLBP=61.4±9.8 years old	Healthy=170.1 cm±9.5 25.6 kg/m^2^±2.4 CLBP=166.7 cm±6.7 26 kg/m^2^±6.6	CCT	9 with CLBP 9 without CLBP	Self-selected walking speed	sEMG	ES, RA, IO, and L	5
Lamoth et al (2006)^24^	Effects of chronic low back pain on trunk coordination and back muscle activity during walking: changes in motor control	17 men 16 women Healthy=31 years old CLBP=38 years old	Healthy=180 cm 72.5 kg CLBP=173 cm 74.4 kg	CCT	19 with CLBP 14 without CLBP	Self-selected and then 1.4-7 km/h	sEMG	ES	5
Poon (2008)^32^	Gait analysis of lumbar muscle activation patterns during constant speed locomotion using surface electromyography	9 men 4 women Healthy=29.8±6.5 years old CLBP=39±12 years old	Healthy=177.1 cm±7.04 70 kg±11.7 CLBP=171.8 cm±3.3 71.5 kg±4.1	CCT	4 with CLBP 9 without CLBP	4.5 km/h and 9 km/h	sEMG	ES and M	8
van der Hulst et al (2010)^17^	Lumbar and abdominal muscle activity during walking in subjects with chronic low back pain: support of the “guarding” hypothesis?	49 Men 47 Women Healthy=40±11 years old CLBP=41±11 years old	Healthy=25 kg/m^2^ CLBP=26 kg/m^2^	CCT	63 with CLBP 33 without CLBP	1.4-5.4 km/h	sEMG	ES, RA, and EO	5

Abbreviations: BF, biceps fermoris; CCT, case control trial; ES, erector spinae; EO, external oblique; GA, gastrocnemius medialis; GM, gluteus medius; GMA, gluteus maximus; HF, hamstring fermoris; IO, internal oblique; L, longissimus; LD, latissimus dorsi; M, multifidus; RA, rectus abdominis; TA, tibialis anterior; VL, vastus lateralis.

### Data analysis

Muscle activation results were extracted from the relevant studies with the mean, standard deviation, and sample size also included. This information was then imported into the spreadsheet of the Rev Man 5.4 software^b^ to carry out the meta-analysis. This systematic review adhered to the PRISMA Checklist and the funneling of review papers were included using the PRISMA flowchart. The final papers selected were then scored using the Physiotherapy Evidence Database (PEDro scale). Finally, a review manager: Rev Man 5.4 software^b^ was used to produce the report and conduct the meta-analysis. An Independent samples *t* test between the 7 healthy groups and the 7 patient groups was performed using the Statistical Package for the Social Sciences.^c^

### Data items

Information extracted from the studies included (i) the characteristics of participants (age, mass, height, history of CLBP); (ii) characteristics of the study (experimental design, sample size, type of EMG used, muscles analyzed, speed of forward propulsion); and (iii) outcomes of the study (muscle activation levels of the erector spinae).

### Risk of bias in individual studies

To assess the risk of bias and methodological quality in the case control trial, the PEDro Scale was used. The PEDro Scale Questionnaire consists of 11 questions that grade the study from a low score (poor) to a high score (excellent). These outputs are presented in the results section.

### Summary measures

The standardized mean difference was used in the meta-analysis to measure effect size. This was highlighted by Liberati et al.^33^ The primary outcome was muscle activation levels in the erector spinae during walking in individuals with and without CLBP. The mean and standard deviation of the outcome measures in each of the studies was used to calculate the 95% confidence interval. I^2^ was calculated to identify heterogeneity between studies. A value close to zero would suggest no heterogeneity. Significant difference was set to *P*<.05.

### Synthesis of results

The heterogeneity statistic I^2^ was placed into categories: no/low, which is less than 25%, moderate, which is between 25% and 50% and finally higher than 50%, which is high between study heterogeneity. This statistic was calculated using the Rev Man 5.4 software (Cochrane Rev Man, Copenhagen, Denmark) and is presented in the results section. There was 1 fixed effect meta-analysis that assessed healthy individuals’ muscles activation compared with individuals that suffer from CLBP. When heterogeneity was less than 25%, the researcher used a fixed effect meta-analysis as the studies were regarded as homogeneous.^34^

### Risk of bias across studies

A funnel plot is provided in the results section as this visually shows whether there was any publication bias. This provides a visual interpretation determining if the data points were asymmetrical.

## Results

### Study selection

On completion of the database search, 604 studies were identified (fig 1). All duplicate studies were removed so that the search outputs were reduced to 481 studies. These remaining studies were then screened by reading the titles and abstracts to confirm if they met the inclusion criteria. Upon completion of this task 60 studies were deemed suitable. A further evaluation was then performed where the full texts of the remaining papers were assessed for eligibility, which meant 18 remained for the final quantitative synthesis. However, only 7 were used for the systematic review and meta-analysis. These studies were case control trials and were selected for the meta-analysis because the sample sizes consisted of both participants with CLBP and without CLBP.

### Study characteristics

Seven were case control trials. The participants’ ages ranged from 18 to 65 years old with 14 of the studies including both sexes and the remaining 4 studies only including men participants. All studies used a treadmill for forward propulsion; however, 10 studies allowed the participants to move at a self-selected walking speed for their own comfort. The other 8 studies used varying speeds however these values were never above 9 km/h. sEMG was the most common EMG test for measuring muscle activation with 3 studies using intramuscular fine wire electrodes. Specifically, 2 studies used fine wire needled EMG while 1 study used both methods. Overall, from the 7 case control studies, 241 subjects were investigated in the review (135 with CLBP and 106 without lower back pain). All studies assessed the erector spinae muscles with some additional trunk muscles being measured (table 1).

### Risk of bias in individual studies

Table 2 shows the risk of bias in each study when compared against the PEDro scale (University of Maastricht, Maastricht, Netherlands). This consisted of 11 questions that aimed to provide a simple score that placed a study at either a low or high risk of publication bias. Each studies’ answer can be seen at each of the questions with an overall score provided. The PEDro score ranged from 4 to 8 for the systematic review outputs and the meta-analysis case control study outputs.

**Table 2:** PEDro scale literature search outputs

Study	Eligibility Criteria	Randomly Allocated	Concealed Allocation	Group Similar at Baseline	Subject Blinding	Therapist Blinding	Assessor Blinding	More Than 85% of Subjects Allocated	Intention to Treat Analysis	Between Group Statistical Analysis	Point Measures and Measures of Variability	PEDro Score
Ansari et al (2018)^28^	Y	N	N	Y	Y	N	N	N	N	Y	Y	5
Diederich (2005)^29^	Y	N	N	N	Y	N	N	N	N	Y	Y	4
Farahpour et al (2018)^30^	Y	N	N	Y	Y	N	N	N	N	Y	Y	5
Hanada et al (2011)^31^	Y	N	N	Y	Y	N	N	N	N	Y	Y	5
Lamoth et al (2006)^24^	Y	N	N	Y	Y	N	N	N	N	Y	Y	5
Poon (2008)^32^	Y	N	Y	Y	Y	Y	Y	N	N	Y	Y	8
van der Hulst et al (2010)^17^	Y	N	N	Y	Y	N	N	N	N	Y	Y	5

Abbreviations: N, no; Y, yes.

### Synthesis of results

The total standardized mean difference between healthy vs CLBP cohorts was 0.48 (in % maximum voluntary isometric contraction) (95% confidence interval=0.21-0.74; *P*<.0001) with the heterogeneity being I^2^=0% (*P*=.890). Farahpour et al^30^ showed the greatest standardized mean difference (0.96; 95% CI=0.20-1.72). This was followed by Hanada et al^31^ (0.54; 95% CI=-0.40-1.49). Diederich^29^ showed the lowest standardized mean difference (0.21; 95% CI=-1.11-1.53). The EMG outputs showed significant differences in activation levels between the healthy and CLBP cohorts (*P*<.001). Overall, based on the small (<0.5) standard mean difference reported in the meta-analysis stratified by the erector spinae activation levels for healthy and CLBP patients; the healthy controls were adequately matched.

### Risk of bias across studies

**Fig 2:**
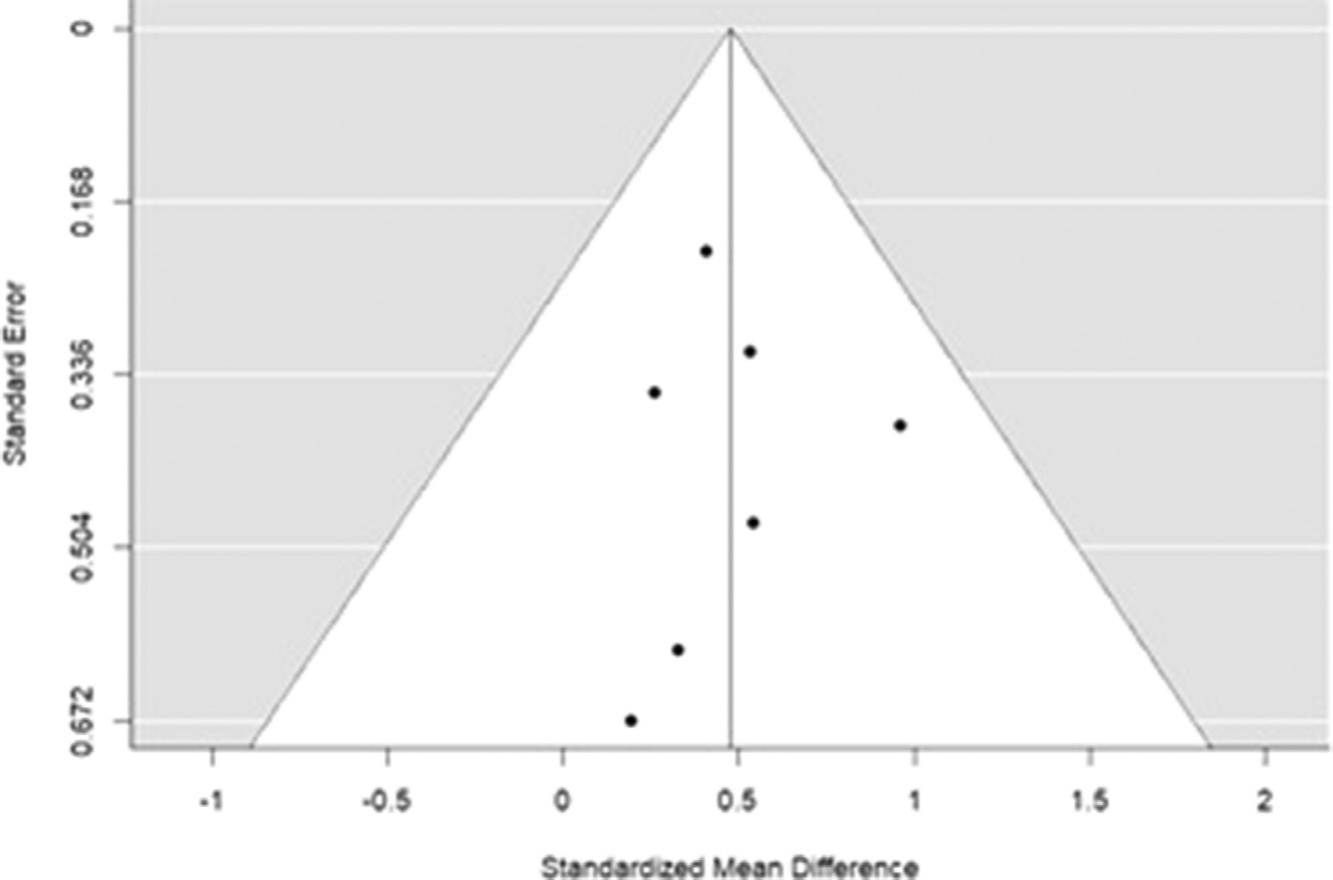
Funnel plot (meta-funnel) for standard mean difference on erector spinae muscle activation levels for healthy and chronic lower back pain patients. Each dot represents a single study with its corresponding standardized mean difference (X-axis) and its associated standard error (Y-axis). Large high powered studies are placed toward the top, and smaller low powered studies toward the bottom. No studies fell outside the funnel plot. Note: Ideally the plot should resemble a pyramid or inverted funnel, with scatter due to sampling variation.

Figure 2 shows a visual representation of the risk of publication bias in the studies involved in the meta-analysis.

**Fig 3:**
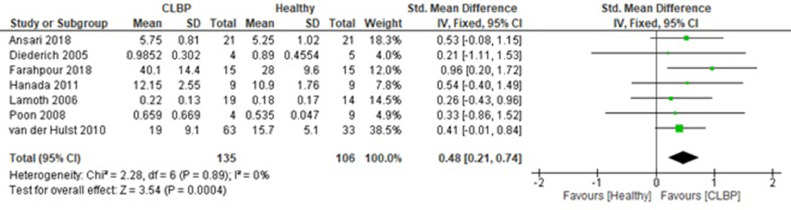
Result summary of meta-analysis stratified by the erector spinae activation levels for healthy and chronic lower back pain patients.

In figure 3, the mean values with respect to each group vary but are not significantly different (*P*=.718). The standard mean difference is small (0.48 [0.21, 0.74]) and so is the mean difference (0.07 [-0.03, 0.17]). Only 3 out of 7 studies produced equal number of sample sizes between the healthy participants and CLBP patients. Overall, in total, there were 135 CLBP patients in comparison with 106 healthy participants.

The summary table (fig 3) shows the outcomes for each intervention group activation levels. Regarding the demographic characteristic of participants, Ansari et al (2018) showed no significant differences between the healthy participants and CLBP patients in terms of age, height and weight (*P*>.05); however, significant differences were observed for the body mass index (BMI) variable.^28^ In Diederich's study,^29^ a total of 9 volunteers met the entrance criteria; however, a combined demographics was reported for both the healthy and CLBP groups. Farahpour et al^30^ and Poon's study in 2008^32^ presented no significant between group differences on age, height, mass, and BMI (*P*>.05). Similarly, Hanada et al^31^ found no significant differences between the healthy and CLBP groups for age, height, and BMI (*P*>.05). Although Lamoth et al^24^ reported no significant differences between the healthy and CLBP groups for weight and height (*P*>.05), age differed between the 2 groups (*P*<.02). van der Hulst et al^17^ showed that the healthy and CLBP groups were comparable in age BMI and sex (*P*>.05). However, the groups differed in educational level and work status (*P*<.01).

## Discussion

This systematic review and meta-analysis provide an overview of recent research, which evaluated the use of EMG to distinguish erector spinae muscle activation during forward propulsion in healthy and CLBP cohorts. The findings from the systematic review focused on 7 case control trials. The systematic review findings suggest that during forward propulsion the CLBP cohorts showed significantly increased EMG activity (especially in the swing phase) when compared with healthy participants. Walking speed influenced muscle activation. At increasing walking speeds even though mean amplitudes increased generally, muscle activation patterns in terms of phase-dependent activations were similar. In terms of the risk of bias from the individual studies, the PEDro score ranged from fair to good for the systematic review outputs and the meta-analysis case control study outputs.

Results from the meta-analysis suggest that muscle activity of the erector spinae muscles was higher in CLBP cohorts when compared with a healthy group. Despite the growing prevalence of LBP with respect to muscle activation and walking, there were only 7 studies that could be used in the meta-analysis. This limited number of studies suggests how relevant and important this systematic review and meta-analysis is. This also highlights research limitations as well as the uncertainty of defining CLBP and a lack of universal methods to follow affected numbers.

### Summary of main results

The meta-analysis results outlined that the total standardized mean difference between healthy vs CLBP was 0.48 (95% confidence interval=0.21-0.74; *P*=.0004) with the heterogeneity being I^2^=0% (*P*=.89). EMG outputs showed significant differences in activation levels between healthy and CLBP cohorts (*P*=.0004). van der Hulst et al^17^ found that erector spinae muscle activation in CLBP cohorts was greater in all aspects of the stride movement during walking. Recruitment of the CLBP patients was dissimilar across the 7 studies. While all 7 studies composed of lower back pain patients, recruitment of CLBP patients were from local clinics; communities including universities and retirement homes; and cooperating exercise therapy practices. Across studies, the results showed small changes in the muscle activation measurements for both the CLBP patients and the healthy comparators. This small variation in the mean values may be attributed to the differences in walking and running speeds that ranged from a self-selected walking speed to a maximum running speed of 9 km/h.

Muscle activation was measured using sEMG across all 7 studies. However, all 7 papers reported using different EMG systems/sensors/surface electrodes. Six studies reported muscle activation measured and recorded at the Erector Spinae while 1 study reported muscle activation at the Multifidus muscle. Anatomically, the Multifidus muscle is a deep back muscle that is part of the Erector Spinae group. Erector spinae muscle activation was determined uniformly across studies; however, differences across the studies may have been influenced by factors such as walking and running duration, EMG type, muscles activation strategy attributed to fatigue, and possibly changes to the muscle recruitment strategy brought about by the variation in the muscle structures of both the CLBP and healthy participants.

### Erector spinae muscle activation levels in CLBP and healthy cohorts

There was greater activation in the erector spinae muscle in CLBP participants as opposed to healthy cohorts. In studies by Wallwork et al^35^ and Arendt-Nielsen et al,^36^ they noted that erector spinae activity was greater during the total stride and the swing phase in CLBP patients and was 8%-48% higher compared with healthy participants.^37^ This result was expected as previous research suggests that this happens for a number of reasons. One reason is the back-extensor muscles showing higher levels of activation to overcome spinal instability.^38^ Lamoth et al^24^ investigated the ability of 2 cohorts’ to adapt their trunk and pelvic rotations and erector spinae activity to unexpected changes in the speed of a treadmill. In this study, the CLBP cohort were less able to adapt the trunk- pelvis coordination and erector spinae activity. This may be an attempt to stabilize the spine and prevent damage to the body. A further reason for this outcome was suggested by Sherman who stated that the reaction resulting in increased muscle activity is associated with muscle spasms.^39^ This finding is supported in a study by Kravitz et al^40^ who stated that participants suffering from lower back pain were able to tense their muscles more than healthy controls, thus by squeezing the muscle an increased muscle activity is achieved. In another study, Hoyt et al^41^ also suggested that during the standing position sEMGs recorded from low back pain sufferers were up to one-third greater and exhibited more tension.

### Types of muscles, electromyography, and crosstalk

One study was unable to provide the full text in English. Sixteen studies analyzed the erector spinae muscles with a large majority also examining the multifidus. Fifteen of the studies used sEMG, 2 used fine wire needled EMG, and 1 used both these methods. Most studies measured the erector spinae at L1-L5 area with L3 being favored. sEMG records the muscle activity on the surface above the muscle on the skin, whereas intramuscular fine wire electrodes are invasive and require the insertion of the needle, which is laborious and requires professional skill.^42^ Intramuscular EMGs, however, are considered to be more reliable in terms of examining deep layer muscles as they can gather a greater variety of frequencies, collect less interfered signals, and as a result, be more sensitive when detecting muscle fatigue.^43^ Intramuscular EMGs have higher reproducibility and reliability rates in comparison with non-invasive methods such as sEMGs when examining deep layer muscles.^44^ This type of EMG also moves with the muscle under the skin during forward propulsion and the procedure can be painful for the participants.^45^ sEMG can be less sensitive when analyzing deep layer muscles because of the overlying musculature, which can generate crosstalk, but is more comfortable for the subject.

Crosstalk is defined as an EMG signal that is generated by a nearby, non-active muscle.^46^ Crosstalk signals can be distinguished from the activity of interest and removed; however, if this is done incorrectly it can lead to incorrect conclusions and misdiagnosis.^47^ Although the evidence indicates that intramuscular EMGs are more reliable than most studies using sEMG, sEMG is often preferred because of its non-invasive methodology. Péter et al^48^ reported that the reliability of sEMGs was comparable with intramuscular EMGs and demonstrated that they were suitable instruments for measuring muscle activity in the lower limbs at self-selected walking speeds. Furthermore, Chapman et al^49^ found that surface electrodes picked up more activity in the adjacent longissimus muscles than the multifidus. Mohseni Bandpei et al^50^ conducted a systematic review that investigated the reliability of sEMG in paraspinal muscle fatigue in healthy and CLBP cohorts. The findings revealed that sEMG was a reliable method for assessing paraspinal muscle fatigue especially in median frequencies.

### Clinical suggestions for research and practice

Physiotherapists involved in rehabilitation programs are often asked to treat lower back pain in patients. This would often include exercises such as stretching/strengthening exercises, and motor control exercises with cardiovascular exercise used sparingly for treatment.^30^ The importance of aerobic exercises such as walking is often overlooked to reduce pain and dysfunction in CLBP patients.^51^ Because of the low effect of walking when compared with running, walking activity makes it an ideal exercise for improving mobility, stability, and cardiovascular fitness. Tongen and Wunderlich^52^ state that jogging produces higher peak forces and shorter contact times compared with walking, which may cause discomfort for a CLBP patient. The erector spinae play an important role in keeping the body upright during forward propulsion and low intensity walking should complement this muscle involvement. A variation of this would be backward walking, which has gained significant interest in recent years and has been suggested to reduce lower back pain levels. Backward walking uses shorter stride lengths and more cadence,^53^ which means there is more muscle activity for less effort.^54^ Backward walking is beneficial because it improves hamstring function, which is often tight in CLBP patients, in conjunction with low back flexibility.^55^ There is published research that has been identified in the literature comparing forward and backward walking. However, as only 1 paper could be identified that compared forward to backward walking, more research is needed in this area. Further to this, no papers that used participants as their own controls could be identified. sEMG can be used to identify injury susceptibility by detection of muscle imbalance.^56^^,^^57^ According to Capin et al,^58^ differences of 15% in muscle strength between limbs can identify potential injury risks as this gives better insight into individual strength, flexibility, and mobility. This can be used to provide objective information, which may be useful in deciding treatment for specific individuals.^59^ According to Chambers and Sutherland,^60^ EMG can be used to provide a baseline for medical interventions and performing post treatments to investigate treatment outcomes and success. Future research should include tools that can better classify and identify lower back pain in patients. Furthermore, studies using large sample sizes should be performed to better compare the activation levels of the erector spinae muscles in healthy and CLBP cohorts. Finally, a more standardized protocol for measuring erector spinae muscle activation during forward propulsion would help to make studies more comparable for future research.

### Limitations

One limitation of this meta-analysis is the small sample size. Although this affects the reliability of the results, 7 research outputs are sufficient in providing a greater, preliminary understanding of the topic area. However, extra investigations would add further significance to the results of this study. Different methodologies used in the studies have proven to be an experimental limitation in this review. For example, studies did not report treadmill incline/velocity, foot wedging, and finally MVC scores and methods. These omissions prevented the use of data that was reported as missing or not included in these studies being compared with full data sets and therefore limited the number of studies that could be included.

Because of the invasive nature of EMGs using needle methods, most of the studies used sEMGs. sEMGs may not be valid for reporting the activity of the erector spinae muscles due to crosstalk.^61^ One potential method of minimizing this was to ensure that participants under investigation have low levels of adipose tissue, which effects recording. This was suggested in a study by Kuiken et al,^62^ which found a reduction in the fat layer reduced EMG crosstalk by 68%. However, because of variations in body composition parameters between subjects, this is not always possible.

## Conclusions

The systematic review identified 7 papers that matched the stated search criteria. These 7 papers were case control trials and were included in the meta-analysis. The sample sizes were reasonably matched and so were the demographics. The case control trials included provided a control and CLBP individuals group with means and standard deviations. The meta-analysis stated that the total standardized mean difference between healthy vs CLBP was 0.48 (95% confidence interval=0.21-0.74; *P*=.0004) with the heterogeneity being I^2^=0% (*P*=.89). The EMG outputs showed significant differences in activation levels between the healthy and CLBP cohorts (*P*=.0004). We can conclude that muscle activation levels are higher in CLBP cohorts compared with healthy populations. Despite this systematic review and meta-analysis highlighting this finding, only 7 case control trials studies were relevant toward addressing our study aim. The limited number of studies highlights the relevance and importance of this topic from a systematic review and meta-analysis perspective. Therefore, for a more definitive conclusion to be obtained, the findings from this study indicate that further case control studies need to be completed to investigate and expand this research area. Lastly, it would be desirable that these studies use larger sample sizes than existing studies.

## Suppliers

a. EndNote X9; Endnote.

b. Rev Man 5.4; Cochrane Rev Man.

c. SPSS 25.0; IBM.

## References

[1] Trompeter K, Fett D, Platen P (2017). Prevalence of back pain in sports: a systematic review of the literature. Sports Med.

[2] Tidy C, Knott L. Lower back pain 2020. Available at: https://patient.info/bones-joints-muscles/back-and-spine-pain/lower-back-pain. Accessed October 16, 2020.

[3] Hoy D, March L, Brooks P (2014). The global burden of low back pain: estimates from the Global Burden of Disease 2010 study. Ann Rheum Dis.

[4] Dionne CE, Dunn KM, Croft PR (2006). Does back pain prevalence really decrease with increasing age? A systematic review. Age Ageing.

[5] Katz JN (2006). Lumbar disc disorders and low-back pain: socioeconomic factors and consequences. J Bone Joint Surg Am.

[6] van Tulder MW, Malmivaara A, Esmail R, Koes BW (2000). Exercise therapy for low-back pain. Cochrane Database Syst Rev.

[7] Hayden JA, Cartwright JL, Riley RD (2012). Exercise therapy for chronic low back pain: protocol for an individual participant data meta-analysis. Syst Rev.

[8] Suh JH, Kim H, Jung GP, Ko JY, Ryu JS (2019). The effect of lumbar stabilization and walking exercises on chronic low back pain: a randomized controlled trial. Medicine (Baltimore).

[9] Roy BA, Vanichkachorn G (2013). Low back pain. ACSMs Health Fit J.

[10] Agten A, Verbrugghe J, Stevens S (2018). Feasibility, accuracy and safety of a percutaneous fine-needle biopsy technique to obtain qualitative muscle samples of the lumbar multifidus and erector spinae muscle in persons with low back pain. J Anat.

[11] Feipel V, De Mesmaeker T, Klein P, Rooze M (2001). Three-dimensional kinematics of the lumbar spine during treadmill walking at different speeds. Eur Spine J.

[12] Palastanga N, Field D, Soames R (2006).

[13] Palastanga N, Soames R (2011). https://books.google.co.uk/books?hl=en&lr=&id=ySwhKi1qmlAC&oi=fnd&pg=PP1&dq=palastanga&ots=YTyYvlACnv&sig=ewBvKKck9IJiIKof40_plC1alfo&redir_esc=y#v=onepage&q=palastanga&f=false.

[14] Biagetti G, Crippa P, Falaschetti L, Orcioni S, Turchetti C (2018). Human activity monitoring system based on wearable sEMG and accelerometer wireless sensor nodes. Biomed Eng Online.

[15] Feger MA, Donovan L, Hart JM, Hertel J (2014). Lower extremity muscle activation during functional exercises in patients with and without chronic ankle instability. PM R.

[16] Xiao J, Gao J, Wang H, Liu K, Yang X (2015). The surface EMG characteristics between erector spinae and vastus lateralis during bending forward and squatting down tasks. Physiol J.

[17] van der Hulst M, Vollenbroek-Hutten MM, Rietman JS, Hermens HJ (2010). Lumbar and abdominal muscle activity during walking in subjects with chronic low back pain: support of the “guarding” hypothesis?. J Electromyogr Kinesiol.

[18] Watson PJ, Booker CK, Main CJ (1997). Evidence for the role of psychological factors in abnormal paraspinal activity in patients with chronic low back pain. J Musculoskelet Pain.

[19] Geisser ME, Haig AJ, Wallbom AS, Wiggert EA (2004). Pain-related fear, lumbar flexion, and dynamic EMG among persons with chronic musculoskeletal low back pain. Clin J Pain.

[20] CDC (2020). https://www.cdc.gov/physicalactivity/basics/age-chart.html.

[21] Bravata DM, Smith-Spangler C, Sundaram V (2007). Using pedometers to increase physical activity and improve health: a systematic review. JAMA.

[22] He LI, Wei WR, Can Z (2018). Effects of 12-week brisk walking training on exercise blood pressure in elderly patients with essential hypertension: a pilot study. Clin Exp Hypertens.

[23] Nielson KA, Radtke RC, Jensen RA (1996). Arousal-induced modulation of memory storage processes in humans. Neurobiol Learn Mem.

[24] Lamoth CJ, Meijer OG, Daffertshofer A, Wuisman PI, Beek PJ (2006). Effects of chronic low back pain on trunk coordination and back muscle activity during walking: changes in motor control. Eur Spine J.

[25] Butowicz CM, Acasio JC, Silfies SP, Nussbaum MA, Hendershot BD (2019). Chronic low back pain influences trunk neuromuscular control during unstable sitting among persons with lower-limb loss. Gait Posture.

[26] Cai C, Yang Y, Kong PW (2017). Comparison of lower limb and back exercises for runners with chronic low back pain. Med Sci Sports Exerc.

[27] Popescu A, Lee H (2020). Neck pain and lower back pain. Med Clin.

[28] Ansari B, Bhati P, Singla D, Nazish N, Hussain ME (2018). Lumbar muscle activation pattern during forward and backward walking in participants with and without chronic low back pain: an electromyographic study. J Chiropract Med.

[29] Diederich JM (2005). https://scholar.google.com/scholar?hl=en&as_sdt=0%2C5&q=Diederich+JM.+Estimate+of+muscle+contribution+to+spinal+loads+during+continuous+passive+motion+for+low+back+pain.+2005.&btnG.

[30] Farahpour N, Jafarnezhadgero A, Allard P, Majlesi M (2018). Muscle activity and kinetics of lower limbs during walking in pronated feet individuals with and without low back pain. J Electromyogr Kinesiol.

[31] Hanada EY, Johnson M, Hubley-Kozey C (2011). A comparison of trunk muscle activation amplitudes during gait in older adults with and without chronic low back pain. PM R.

[32] Poon WM (2008). https://researchrepository.rmit.edu.au/esploro/outputs/graduate/Gait-analysis-of-lumbar-muscle-activation-patterns-during-constant-speed-locomotion-using-surface-electromyography/9921861590501341.

[33] Liberati A, Altman DG, Tetzlaff J (2009). The PRISMA statement for reporting systematic reviews and meta-analyses of studies that evaluate healthcare interventions: explanation and elaboration. BMJ.

[34] K Ried (2006). https://scholar.google.com/scholar?hl=en&as_sdt=0%2C5&q=Ried+K.+Interpreting+and+understanding+meta-analysis+graphs%3A+a+practical+guide.+2006&btnG=.

[35] Wallwork TL, Stanton WR, Freke M, Hides JA (2009). The effect of chronic low back pain on size and contraction of the lumbar multifidus muscle. Man Ther.

[36] Arendt-Nielsen L, Graven-Nielsen T, Svarrer H, Svensson P (1996). The influence of low back pain on muscle activity and coordination during gait: a clinical and experimental study. Pain.

[37] Tsao H, Hodges PW (2008). Persistence of improvements in postural strategies following motor control training in people with recurrent low back pain. J Electromyogr Kinesiol.

[38] Vogt L, Pfeifer K, Banzer W (2003). Neuromuscular control of walking with chronic low-back pain. Man Ther.

[39] Sherman RA (1985). Relationships between strength of low back muscle contraction and reported intensity of chronic low back pain. Am J Phys Med.

[40] Kravitz E, Moore ME, Glaros A (1981). Paralumbar muscle activity in chronic low back pain. Arch Phys Med Rehabil.

[41] Hoyt WH, Hunt HH, De Pauw MA (1981). Electromyographic assessment of chronic low-back pain syndrome. J Am Osteopath Assoc.

[42] DeLuca P, Bell K, Davis R (1997). Using surface electrodes for the evaluation of the rectus femoris, vastus medialis and vastus lateralis muscles in children with cerebral palsy. Gait Post.

[43] Davis BA, Krivickas LS, Maniar R, Newandee DA, Feinberg JH (1998). The reliability of monopolar and bipolar fine-wire electromyographic measurement of muscle fatigue. Med Sci Sports Exerc.

[44] Merletti R, Farina D (2009). Analysis of intramuscular electromyogram signals. Philos Trans A Math Phys Eng Sci.

[45] Hodges PW, Gandevia SC (2000). Pitfalls of intramuscular electromyographic recordings from the human costal diaphragm. Clin Neurophysiol.

[46] Farina D, Merletti R, Indino B, Graven-Nielsen T (2004). Surface EMG crosstalk evaluated from experimental recordings and simulated signals. Reflections on crosstalk interpretation, quantification and reduction. Methods Inf Med.

[47] Van Vugt J, Van Dijk J (2001). A convenient method to reduce crosstalk in surface EMG. Clin Neurophysiol.

[48] Péter A, Andersson E, Hegyi A (2019). Comparing surface and fine-wire electromyography activity of lower leg muscles at different walking speeds. Front Physiol.

[49] Chapman AR, Vicenzino B, Blanch P, Knox JJ, Hodges PW (2010). Intramuscular fine-wire electromyography during cycling: repeatability, normalisation and a comparison to surface electromyography. J Electromyogr Kinesiol.

[50] Mohseni Bandpei MA, Rahmani N, Majdoleslam B, Abdollahi I, Ali SS, Ahmad A (2014). Reliability of surface electromyography in the assessment of paraspinal muscle fatigue: an updated systematic review. J Manipulative Physiol Ther.

[51] Chatzitheodorou D, Kabitsis C, Malliou P, Mougios V (2007). A pilot study of the effects of high-intensity aerobic exercise versus passive interventions on pain, disability, psychological strain, and serum cortisol concentrations in people with chronic low back pain. Phys Ther.

[52] Tongen A, Wunderlich RE. Biomechanics of running and walking. Mathematics and Sports 2010;43:1-12 https://books.google.co.uk/books?hl=en&lr=&id=WkqMPCVmF8oC&oi=fnd&pg=PA315&dq=A+Tongen+and+RE+Wunderlich,+Biomechanics+of+running+and+walking,+Mathematics+and+Sports,+43,+2010,+1–12.&ots=yL4X2dpT-b&sig=jVvNAv9AwmuhqEBqy8O0hUkzkKY&redir_esc=y#v=onepage&q&f=false.

[53] Vilensky JA (1987). A kinematic comparison of backward and forward walking in humans. J Hum Move Stud.

[54] Gray G (1990). Successful strategies for closed chain testing and rehabilitation. Chain Reaction, Adrian M..

[55] Whitley CR, Dufek JS (2011). Effects of backward walking on hamstring flexibility and low back range of motion. Int J Exerc Sci.

[56] Croisier JL, Ganteaume S, Binet J, Genty M, Ferret JM (2008). Strength imbalances and prevention of hamstring injury in professional soccer players: a prospective study. Am J Sports Med.

[57] Fousekis K, Tsepis E, Poulmedis P, Athanasopoulos S, Vagenas G (2011). Intrinsic risk factors of non-contact quadriceps and hamstring strains in soccer: a prospective study of 100 professional players. Br J Sports Med.

[58] Capin JJ, Zarzycki R, Arundale A, Cummer K, Snyder-Mackler L (2017). Report of the primary outcomes for gait mechanics in men of the ACL-SPORTS trial: secondary prevention with and without perturbation training does not restore gait symmetry in men 1 or 2 years after ACL reconstruction. Clin Orthopaed Relat Res.

[59] Marín J, Blanco T, Marín JJ, Moreno A, Martitegui E, Aragüés JC (2019). Integrating a gait analysis test in hospital rehabilitation: a service design approach. PLoS One.

[60] Chambers HG, Sutherland DH (2002). A practical guide to gait analysis. J Am Acad Orthop Surg.

[61] Panjabi MM (2003). Clinical spinal instability and low back pain. J Electromyogr Kinesiol.

[62] Kuiken TA, Lowery MM, Stoykov NS (2003). The effect of subcutaneous fat on myoelectric signal amplitude and cross-talk. Prosthet Orthot Int.

